# Evaluation of severe hypoglycemia and common mental disorders in patients receiving insulin analogues for treatment of type 1 diabetes

**DOI:** 10.20945/2359-3997000000315

**Published:** 2020-12-15

**Authors:** Gabriela Berlanda, Gabriela H. Telo, Barbara Côrrea Krug, Rafael Selbach Scheffel, Bruna Pasinato, Fernando Iorra, João Gabbardo dos Reis, Paulo Dornelles Picon, Beatriz D. Schaan

**Affiliations:** 1 Universidade Federal do Rio Grande do Sul Porto Alegre RS Brasil Programa de Pós-Graduação em Endocrinologia, Universidade Federal do Rio Grande do Sul (UFRGS), Porto Alegre, RS, Brasil; 2 Hospital de Clínicas de Porto Alegre Porto Alegre RS Brasil Hospital de Clínicas de Porto Alegre, Porto Alegre, RS, Brasil; 3 Pontifícia Universidade Católica do Rio Grande do Sul Departamento de Medicina Interna Escola de Medicina Porto Alegre RS Brasil Departamento de Medicina Interna, Escola de Medicina da Pontifícia Universidade Católica do Rio Grande do Sul, Porto Alegre, RS, Brasil; 4 Secretaria Estadual da Saúde do Rio Grande do Sul Porto Alegre RS Brasil Secretaria Estadual da Saúde do Rio Grande do Sul, Porto Alegre, RS, Brasil; 5 Universidade Federal do Rio Grande do Sul Porto Alegre RS Brasil Universidade Federal do Rio Grande do Sul (UFRGS), Porto Alegre, RS, Brasil; 6 Contingência de Combate ao COVID-19 do Governo do Estado de São Paulo São Paulo SP Brasil Coordenação Executiva do Comitê de Contingência de Combate ao COVID-19 do Governo do Estado de São Paulo, São Paulo, SP, Brasil

**Keywords:** Severe hypoglycemia, type 1 diabetes, common mental disorders

## Abstract

This is a retrospective report of the frequency of severe hypoglycemia and the association between common mental disorders and type 1 diabetes mellitus treated with insulin analogues. Patients with severe hypoglycemia compared with those without this complication had a higher prevalence of positive screening for common mental disorders (88% vs.77%, respectively, p = 0.03).

## INTRODUCTION

Intensive treatment of type 1 diabetes (T1D) prevents and slows the progression of long-term complications of the disease, but severe hypoglycemia is a barrier in achieving strict glucose control in these patients (1). Insulin analogues, compared with human insulins, can better mimic endogenous insulin production and possibly contribute to reducing hypoglycemia (2) and increasing patient satisfaction with treatment (3). A bidirectional association between common mental disorders and severe hypoglycemia has been described (4,5), although this concept remains debatable.

The purpose of this study was to evaluate the frequency of severe hypoglycemia and its association with common mental disorders in patients with T1D treated with insulin analogues after introduction of these types of insulin in the public health system in Southern Brazil.

## METHODS

To evaluate the aspects highlighted above, we studied 516 adults with T1D living across 38 cities in Southern Brazil. The patients included in this study were selected from participants in a program of no-cost distribution of insulin analogues, whose enrollment in Brazil requires at least two severe hypoglycemic events within a period of 6 months. Severe hypoglycemia, defined as hypoglycemic episodes requiring assistance from another person, was evaluated using a self-report questionnaire. The eligible population included patients aged 18 years or older who were using short-acting insulin analogues (lispro, aspart, or glulisine) and/or long-acting insulin analogues (glargine, detemir, or degludec). Patients with cognitive deficits or communication barriers were excluded.

Treatment satisfaction was evaluated using the Diabetes Treatment Satisfaction Questionnaire status version (DTSQs). The total DTSQs score varied from 0 to 36, with higher scores indicating greater treatment satisfaction (6).

For mental health screening, the participants filled out the Portuguese version of the 12-item General Health Questionnaire (GHQ-12), previously validated in the Brazilian population (7). The questionnaire is a self-administered screening tool to detect non-psychotic symptoms of mental health disorders in community settings. It checks whether participants recently experienced any specific symptom or behavior on a four-point Likert scale, ranging, per item, from 1 to 4. A score equal to or greater than 3 indicated a positive screening for common mental disorders (8).

The protocol of the present study was approved by the Research Ethics Committee of *Hospital de Clínicas de Porto Alegre* (*Certificado de Apresentação para Apreciação Ética* [CAAE] 1.283.728).

## RESULTS AND DISCUSSION

The patients had a median age of 35 years (interquartile range 28-45 years) and were 52% women. In all, 101 (20%) patients reported severe hypoglycemia in the month before the data collection (Table 1). All patients used multiple daily insulin injections, and none of them used insulin pumps or sensors. Patients with severe hypoglycemia were older, had lower education level and longer diabetes duration, and used beta-blockers more often than those without severe hypoglycemia. Patients with severe hypoglycemia (versus those without this complication) also had a higher prevalence of positive screening for common mental disorders (88% *vs.* 77%, respectively, p = 0.027), as well as more symptoms of depression, anxiety, somatic signs, and social withdrawal. Additionally, the median DTSQs score was lower in patients with severe hypoglycemia compared with those without this complication.

**Table 1:** Baseline patient and disease characteristics stratified according to frequency of severe hypoglycemia

Characteristic	Overall study population (N = 516)	Severe hypoglycemia		p
		No (N = 409)	Yes (N = 101)	
Age (years)	35 (28-45)	34 (27-45)	38 (31-49)	0.016
Sex (% women)	259 (52)	199 (50)	59 (56)	0.446
Ethnicity (% white)	446 (88)	348 (87)	91 (90)	0.552
School (% complete higher education)	189 (37)	162 (40)	27 (26)	0.006
Age at diagnosis (years)	17 (11-27)	17 (11-27)	18 (11-28)	0.664
Diabetes duration (years)	18 (11-25)	17 (10-24)	19 (13-27)	0.012
Duration of use of insulin analogues (years)	5 (3-10)	5 (3-10)	5 (2-10)	0.917
Body mass index (kg/m^2^)	24 (22-27)	24 (22-27)	25 (22-27)	0.688
Beta-blocker use	27 (5)	16(4)	11(10)	0.023
Rapid-acting insulin analogue use	458 (91)	360 (88)	94 (93)	0.439
Long-acting insulin analogue use	431 (86)	340 (83)	85 (84)	0.539
GHQ-12, CMD screening (≥3)	395 (77)	301 (77)	90 (88)	0.027
DTSQs total score	32 (29-35)	32 (29-35)	31 (27-34)	0.007

Data are presented as median (interquartile range) or n (%). GHQ-12 score, General Health Questionnaire screening. The GHQ-12 is considered positive for common mental disorders (CMD) when the score is ≥3 (%patients). DTSQs, Diabetes Treatment Satisfaction Questionnaire. DTSQs total score, items 1,4,5,6,7,8 (range 0-36). ANOVA was used for comparing numerical variables with normal distribution, and the Kruskal-Wallis test for data with a non-normal distribution. The Mann-Whitney and Wilcoxon tests were used for paired samples. Categorical variables were compared using the chi-square test. p<0.05 indicated statistically significant differences between groups.

This study has some limitations. First, severe hypoglycemia is associated with depression (9), and since severe hypoglycemia was also a criterion for enrollment in the government program of distribution of insulin analogues, it may have impacted our findings in this study. Second, the observational nature of the study could lead to reverse causality. Third, the prevalence of severe hypoglycemia was considerably higher compared with previous reports (10,11). This discrepancy may be due to different ways of assessing hypoglycemia and the diversity of the populations studied. Differences in health care delivery and local economic conditions may also affect patient access to education and blood glucose monitoring, which in turn may interfere in the recording of hypoglycemic episodes. Moreover, studies have reported that the use of beta-blockers increases the potential risk of severe hypoglycemia (12), and that psychiatric disorders are associated with an increased number of severe hypoglycemic episodes (9), with possible intentional manipulation of insulin doses by patients (13). The bidirectional association between mental health disorders and severe hypoglycemia may increase the burden of diabetes (4,5) and significantly impact patient satisfaction with treatment (14).
